# Bone regeneration effects of human allogenous bone substitutes: a preliminary study

**DOI:** 10.5051/jpis.2010.40.3.132

**Published:** 2010-06-25

**Authors:** Deok-Won Lee, Ki-Tae Koo, Yang-Jo Seol, Yong-Moo Lee, Young Ku, In-Chul Rhyu, Chong-Pyoung Chung, Tae-Il Kim

**Affiliations:** Department of Periodontology and Dental Research Institute, Seoul National University College of Dentistry, Seoul, Korea.

**Keywords:** Bone Substitutes, Osteogenesis, Transplantation, X-Ray Microtomography

## Abstract

**Purpose:**

The purpose of this study was to compare the bone regeneration effects of cortical, cancellous, and cortico-cancellous human bone substitutes on calvarial defects of rabbits.

**Methods:**

Four 8-mm diameter calvarial defects were created in each of nine New Zealand white rabbits. Freeze-dried cortical bone, freeze-dried cortico-cancellous bone, and demineralized bone matrix with freeze-dried cancellous bone were inserted into the defects, while the non-grafted defect was regarded as the control. After 4, 8, and 12 weeks of healing, the experimental animals were euthanized for specimen preparation. Micro-computed tomography (micro-CT) was performed to calculate the percent bone volume. After histological evaluation, histomorphometric analysis was performed to quantify new bone formation.

**Results:**

In micro-CT evaluation, freeze-dried cortico-cancellous human bone showed the highest percent bone volume value among the experimental groups at week 4. At week 8 and week 12, freeze-dried cortical human bone showed the highest percent bone volume value among the experimental groups. In histologic evaluation, at week 4, freeze-dried cortico-cancellous human bone showed more prominent osteoid tissue than any other group. New bone formation was increased in all of the experimental groups at week 8 and 12. Histomorphometric data showed that freeze-dried cortico-cancellous human bone showed a significantly higher new bone formation percentile value than any other experimental group at week 4. At week 8, freeze-dried cortical human bone showed the highest value, of which a significant difference existed between freeze-dried cortical human bone and demineralized bone matrix with freeze-dried cancellous human bone. At week 12, there were no significant differences among the experimental groups.

**Conclusions:**

Freeze-dried cortico-cancellous human bone showed swift new bone formation at the 4-week healing phase, whereas there was less difference in new bone formation among the experimental groups in the following healing phases.

## INTRODUCTION

Autogenous bone grafts are considered to be the gold standard in bone regeneration because of their osteogenic activity [1]. Due to the donor site morbidity and limited quantity of autogenous bone, however, clinicians and patients have been searching for alternative materials. Human allogenic bone graft materials have been regarded as adequate bone substitutes because of their availability without additional surgery [2]. In 1965, it was reported that the implantation of demineralized bone into extrabony sites led to the formation of bone ossicles [3]. In this process, known as osteoinduction, bone morphogenetic components stimulate undifferentiated mesenchymal cells to become osteoprogenitor cells. It has been subsequently reported that bone morphogenetic proteins were identified in decalcified allograft materials [4,5]. Due to these results, human bone allografts with osteoinductivity have drawn attention as an alternative to autogenous bone.

Human allografts can be classified into cortical, cancellous, and cortico-cancellous allografts according to their source. They can also be classified into freeze-dried bone allografts (FDBA) and decalcified freeze-dried bone allografts (DFDBA), also known as demineralized bone matrix (DBM), according to their decalcification process. There have been many studies comparing the osteoinductive effects of FDBA and DFDBA [6-9]. However, the differences in osteoinductive effects among cortical, cancellous, and cortico-cancellous human bone have not yet been reported. Therefore, this study was performed to compare the bone regeneration effects of human cortical bone, cancellous bone, and cortico-cancellous bone in calvarial defects of rabbits.

## MATERIALS AND METHODS

### Experimental animal preparation

Nine mature New Zealand white male rabbits were used for the experiments. The animal research protocol was approved by the Institute of Laboratory Animal Resources, Seoul National University.

### Surgical procedure

**Figure 1:**
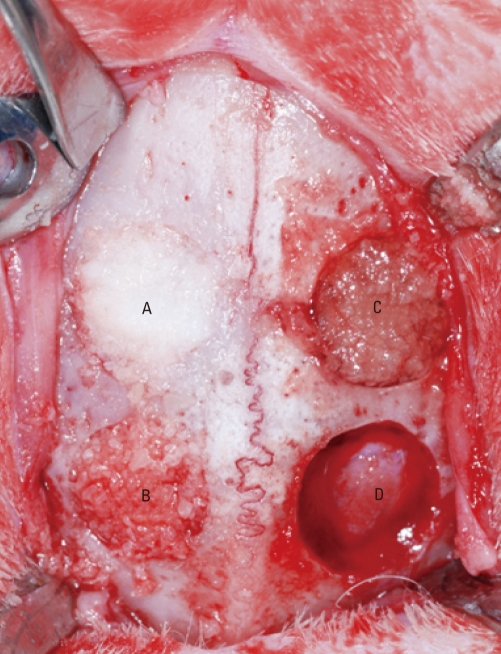
Four 8-mm diameter calvarial defects were created. Freeze-dried cortical human bone (A), freeze-dried cortico-cancellous human bone (B), and demineralized bone matrix with cancellous human bone (C) were inserted into the three experimental defect sites. No biomaterial was grafted into the control defect site (D).

All animals were anesthetized with solution of 91% ketamine hydrochloride (Ketalar™, Yuhan Co., Seoul, Korea) and 9% xylazine (Rumpun™, Bayer Korea Ltd., Seoul, Korea) via an intramuscular injection. After the surgical site was disinfected with betadine, local anesthesia was provided using a 2% lidocaine solution. The calvariae were exposed through a midline incision. The external cortical plates were removed carefully using an 8-mm trephine bur with saline irrigation. Four calvarial defects were created and filled with the human allogenous bone graft materials; freeze-dried cortical human bone powder (SureOss™, Hans Biomed Corp., Seoul, Korea), freeze-dried cortico-cancellous human bone powder (OsteOss™, Hans Biomed Corp., Seoul, Korea), and demineralized bone matrix with cancellous human bone (ExFuse™ II, Hans Biomed Corp., Seoul, Korea). The non-grafted defect was regarded as the control (Fig. 1). The periosteum were closed by using resorbable suture materials (5-0 Vicryl™, Ethicon, Somerville, USA), and the skin was sutured by monofilament suture materials (4-0 Ethilon™, Johnson & Johnson Int., Edinburgh, UK). The suture materials were removed at 10 days postsurgery. The rabbits were sacrificed at 4, 8, or 12 weeks following surgery.

### Micro-computed tomography (micro-CT)

The calvarial bone was taken and fixed in a 10% neutral buffered formalin solution. The retrieved specimens were trimmed and scanned using a high-resolution micro-CT system (SkyScan 1172™, Skyscan, Kontich, Belgium). Scanned data were reconstructed using image analysis software (CT-analyzer™, Skyscan, Kontich, Belgium). The percent bone volume values, which were the percentile ratio of radio-opaque regions of interest over the total defect volume, were measured.

### Histological procedure

After micro-CT scanning, specimens were sectioned and dehydrated in ethanol solutions of increasing concentration. Then, they were decalcified in 5% formic acid and embedded in paraffin. The final sections were cut into 5-µm thicknesses and stained with hematoxylin-eosin for a microscopic evaluation.

### Histomorphometric analysis

The central sections were chosen for histomorphometric analysis. Photographs were taken with light microscope (BH-2™, Olympus Optical, Osaka, Japan). Computer-assisted histomorphometric measurements of the newly formed bone were obtained using an automated image analysis system. (ScopeEye™, Techsan, Seoul, Korea). The new bone formation values, which were the percentile ratio of newly formed bone area over the total defect area, were calculated.

### Statistical analysis

Statistical analysis was performed using statistical analysis software (SPSS™, SPSS Inc., Chicago, USA). Significant differences among groups were identified by one-way ANOVA with Tukey's ad-hoc test. Data were represented as means±SD, and *P* values of 0.05 were considered to be statistically significant.

## RESULTS

### Micro-CT analysis

**Figure 2:**
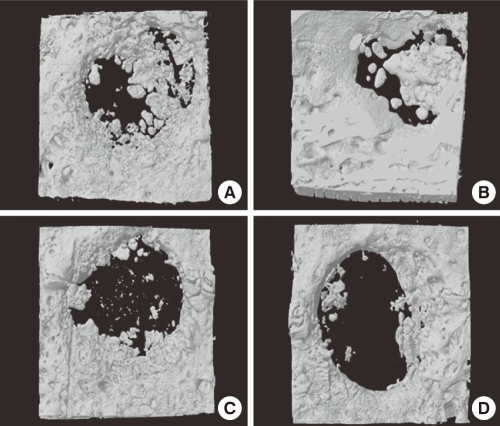
Three dimensional images of freeze-dried cortical human bone (A), freeze-dried cortico-cancellous human bone (B), demineralized bone matrix with cancellous human bone (C), and the control (D) at week 4. Freeze-dried cortico-cancellous human bone (B) revealed greatest amount of newly formed bone.

**Figure 3:**
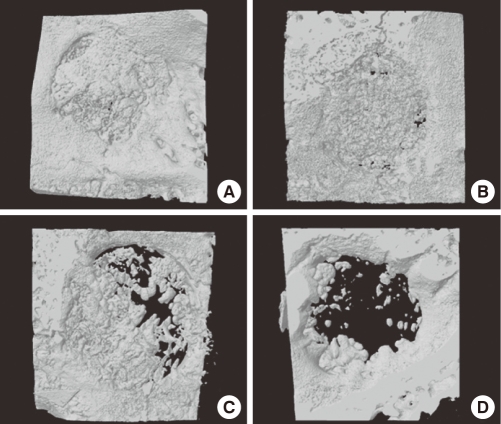
Three dimensional images of freeze-dried cortical human bone (A), freeze-dried cortico-cancellous human bone (B), demineralized bone matrix with cancellous human bone (C), and the control (D) at week 8. Freeze-dried cortical human bone (A) showed better development of trabecular bone than any other group.

**Figure 4:**
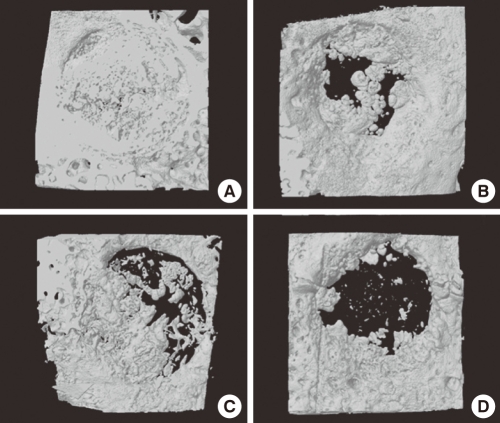
Three dimensional images of freeze-dried cortical human bone (A), freeze-dried cortico-cancellous human bone (B), demineralized bone matrix with cancellous human bone (C), and the control (D) at week 12. The defects were nearly closed by newly formed bone, except for the control.

At week 4, freeze-dried cortico-cancellous human bone showed more new bone formation than any other group. Mostly, new bone formation appeared at the margins of the defect sites. There was only a small amount of new bone formation near the central region (Fig. 2). At week 8, freeze-dried cortical human bone enhanced new bone formation compared to the other experimental groups. More newly formed bone was observed in the central region as well as at marginal sites than at week 4 (Fig. 3). At week 12, the defects were nearly closed by newly formed bone in all of the experimental groups. In the control group, the defect was not closed yet (Fig. 4).

**Figure 5:**
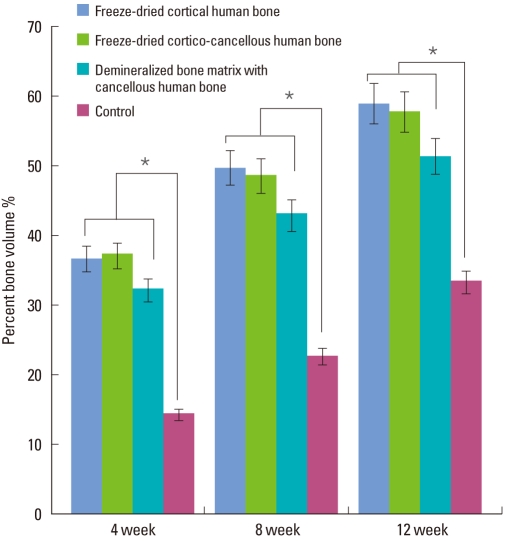
Percent bone volume values of each group from micro-CT analysis. All experimental groups showed significantly higher percent bone volume values than the control group at week 4, 8, and 12. ^*^Significant difference between the experimental and the control group (*P* < 0.05).

All experimental groups showed significantly higher percent bone volume values than the control group at week 4, 8, and 12 (*P* < 0.05). At week 4, although freeze-dried cortico-cancellous human bone (37.12 ± 4.04%) showed a higher value than freeze-dried cortical human bone (36.63 ± 3.20%) or demineralized bone matrix with cancellous human bone (32.18 ± 2.78%), there were no significant differences among the three experimental groups (Fig. 5). At week 8, freeze-dried cortical human bone (49.53 ± 3.73%) showed a higher value than freeze-dried cortico-cancellous human bone (48.64 ± 4.68%) or demineralized bone matrix with cancellous human bone (42.91 ± 2.34%), but there were no significant differences among the three experimental groups (Fig. 5). At week 12, freeze-dried cortical human bone (58.99 ± 1.17%) showed a higher value than freeze-dried cortico-cancellous human bone (57.62 ± 1.10%) or demineralized bone matrix with cancellous human bone (51.33 ± 4.62%), without any significant differences (Fig. 5).

### Histological evaluation

**Figure 6:**
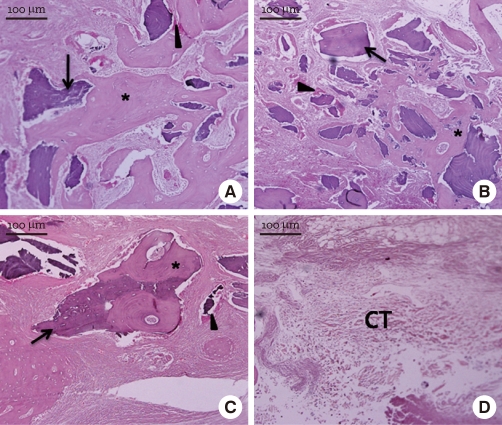
Light micrographs at week 4 (H&E stain). Freeze-dried cortical human bone (A), freeze-dried cortico-cancellous human bone (B), demineralized bone matrix with cancellous human bone (C), and the control (D). All experimental groups showed new bone formation (asterisk) at the margins of the defect sites. Graft materials (arrow) were surrounded by newly formed osteoid tissue (arrow-head). In freeze-dried cortico-cancellous human bone (B), newly formed bone was observed more prominently than in any other group. In the control, loose connective tissue (CT) filled the defect sites.

At week 4, the freeze-dried cortical human bone showed new bone formation at the margins of the defect sites. There was only a small amount of new bone formation near the central region. The freeze-dried cortical human bone substitute particles were surrounded by newly formed osteoid tissue (Fig. 6A). In the freeze-dried cortico-cancellous human bone, prominent osteoid tissue was observed around the small cancellous bone graft particles (Fig. 6B). In the demineralized bone matrix with cancellous human bone, a newly formed bone was observed at the margins of the defect. Osteoid tissue was observed around the graft particles (Fig. 6C). In the control group, newly formed bone was barely observed at the margin of the defect. In the central part, loose connective tissue filled the defect sites (Fig. 6D).

**Figure 7:**
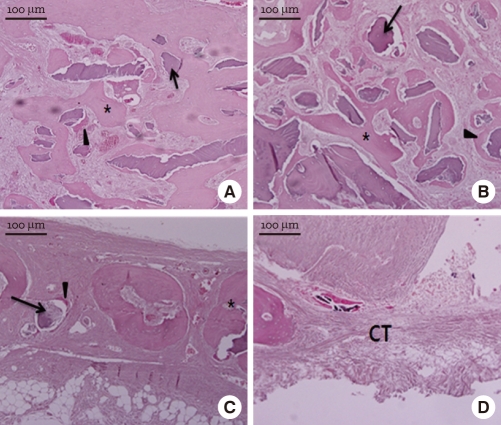
Light micrographs at week 8 (H&E stain). Freeze-dried cortical human bone (A), freeze-dried cortico-cancellous human bone (B), demineralized bone matrix with cancellous human bone (C), and the control (D). The new bone (asterisk) surrounded the graft particles (arrow). There were abundant osteogenic cells and matrix (arrowhead) around the graft particles. In the control, the central area was still filled mainly with loose connective tissue (CT).

At week 8, all of the experimental groups showed newly formed bone in the central region as well as at marginal sites. Figure 7 shows us that the newly formed bone surrounded the graft particles. In the control group, new bone formation was very rare and the defect was mainly filled with loose connective tissue.

**Figure 8:**
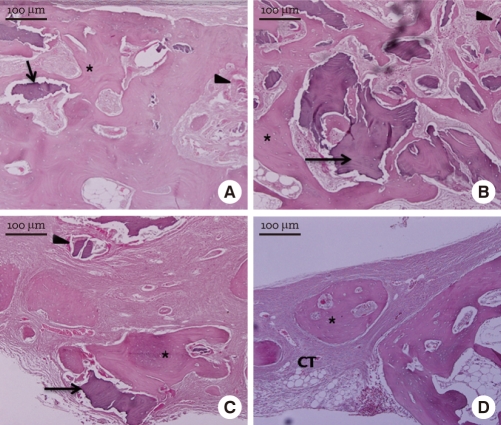
Light micrographs at week 12 (H&E stain). Freeze-dried cortical human bone (A), freeze-dried cortico-cancellous human bone (B), demineralized bone matrix with cancellous human bone (C), and the control (D). The newly formed bone (asterisk) showed a more mature pattern than at week 4 or 8. In the control, loose connective tissue (CT) still filled most of the defect area.

At week 12, the experimental groups showed prominent new bone formation (Fig. 8). The newly formed bone showed a more mature pattern than at week 4 or 8. Also, active bone formation with osteogenic cells and matrix was visible around the graft particles. In the control, loose connective tissue still filled most of the defect area.

### Histomorphometric analysis

**Figure 9:**
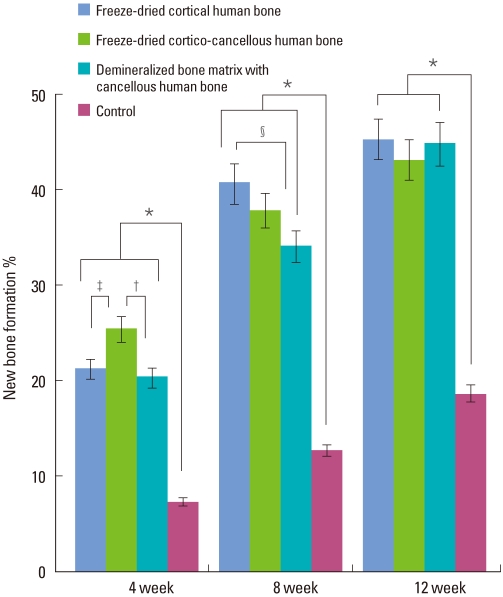
New bone formation values of each group from histomorphometric analysis. All experimental groups showed significantly higher new bone formation values than the control at week 4, 8, and 12. ^*^Significant difference between the experimental and the control groups (*P* < 0.05). ^†^Significant difference between freeze-dried cortico-cancellous human bone and demineralized bone matrix with cancellous human bone (*P* < 0.05). ^‡^Significant difference between freeze-dried cortical human bone and freeze-dried cortico-cancellous human bone (*P* < 0.05). ^§^Significant difference between freeze-dried cortical human bone and demineralized bone matrix having cancellous human bone (*P* < 0.05).

At week 4, all the experimental groups showed significantly higher new bone formation values than the control group (*P* < 0.05). The freeze-dried cortico-cancellous human bone showed a significantly higher new bone formation value (25.37 ± 2.74%) than the freeze-dried cortical human bone (21.15 ± 1.02%) or demineralized bone matrix with cancellous human bone (20.43 ± 1.43%) (*P* < 0.05) (Fig. 9). At week 8, all experimental groups showed significantly higher new bone formation values than the control group (*P* < 0.05). The freeze-dried cortical human bone showed a higher new bone formation value (40.67 ± 1.16%) than the freeze-dried cortico-cancellous human bone (37.77 ± 1.29%) or demineralized bone matrix with cancellous human bone (34.00 ± 3.50%). The difference was significant between the freeze-dried cortical human bone and the demineralized bone matrix with cancellous human bone. At week 12, all experimental groups showed significantly higher new bone formation values than the control group (*P* < 0.05). There were no significant differences in new bone formation values among the freeze-dried cortical human bone (45.26 ± 2.91%), freeze-dried cortico-cancellous human bone (43.02 ± 1.84%), and demineralized bone matrix with cancellous human bone (44.77 ± 2.99%).

## DISCUSSION

It has been reported that demineralized freeze-dried bone showed greater osteogenic effects than freeze-dried bone grafts [10]. On the other hand, it was demonstrated that freeze-dried bone has greater osteoconductivity than demineralized freeze-dried bone [8]. It has also been reported that there was no significant difference in osteogenic effect between freeze-dried and demineralized freeze-dried bone grafted into humans [9,11]. However, these studies were focused on comparing differences between freeze-dried bone and demineralized freeze-dried bone. Therefore, the comparison of the osteogenic effects among cortical, cortico-cancellous, and cancellous human bone is worthwhile. This is the first comparative report on the bone regeneration effect of human allogenous bone grafts from different sources.

In the 4-week healing phase, the freeze-dried cortico-cancellous human bone showed a significantly higher new bone formation value than the freeze-dried cortical human bone or demineralized bone matrix with cancellous human bone according to the histomorphometric results. In addition, in the micro-CT evaluation, freeze-dried cortico-cancellous human bone showed a highest percent bone volume value than any other group, although there was no statistical significance.

Optimal bone graft substitutes should provide structure for revascularization and sufficient mechanical stability [12]. Cancellous bone has been observed to be revascularized more quickly in the graft sites than cortical bone and fast revascularization has been regarded as an essential prerequisite for repopulation of bone forming cells [13-15].

Considering the previous results, it can be inferred that new bone formation proceeds rapidly in cancellous bone, and rapid consolidation occurs with the development of new cortex in cancellous bone. In this study, it can be hypothesized that the presence of cancellous bone in freeze-dried cortico-cancellous human bone graft material was related to the fast revascularization, while the remaining cortical bone seems to be maintaining the stability of the grafted site. The interplay of these two key factors is supposed to correlate with the high osteogenic effects of freeze-dried cortico-cancellous human bone in the 4-week healing phase. Further studies are needed to elucidate the effects of mixing cancellous bone with cortical bone in the future.

According to our histomorphometric results, the freeze dried cortical human bone showed higher new bone formation value than the other human allografts in the 8 and 12 week healing phases. In the micro-CT evaluation, the freeze-dried cortical human bone showed a higher percent bone volume value than any other group. However, there were no significant differences among the experimental groups. These results imply that the difference in the osteogenic effects among the experimental groups was diminished in the late healing period compared to the early healing period. The present result is consistent with previous findings that there was no significant difference in new bone formation between DFDBA and FDBA, which was evaluated at the late healing phase [9,11].

One interesting finding in our study is that freeze-dried cortical human bone caught up with an early phase high osteogenic level of freeze-dried cortico-cancellous human bone and approached a slightly higher ostegenic level as the healing time went on. It can be surmised that this preparation provides an appropriate three-dimensional durable structure to serve as an osteoconductive matrix compared to cancellous bone. This resorption-resistant stable structure can provide a long-lasting framework for slowly proceeding appositional new bone formation. This is compatible with the results that cancellous bone is mostly resorbed within three months, but cortical bone can resist resorption for six months, and even at one year after implantation almost 40% of the original grafted bone remains [14]. Also, it was proposed that this structural stability is especially essential during the remodeling period [16]. From this point of view, special regard should be paid to the role of cortical bone for successful long-term osteogenic results.

In conclusion, in the 4-week healing phase, freeze-dried cortico-cancellous human bone showed faster new bone formation than freeze-dried cotical human bone or demineralized bone matrix with freeze-dried cancellous human bone. However, in the following 8- and 12-week healing phase, there was a smaller difference in new bone formation among the experimental groups than in the early healing phase.
